# Identification of novel regulatory factor X (RFX) target genes by comparative genomics in *Drosophila *species

**DOI:** 10.1186/gb-2007-8-9-r195

**Published:** 2007-09-17

**Authors:** Anne Laurençon, Raphaëlle Dubruille, Evgeni Efimenko, Guillaume Grenier, Ryan Bissett, Elisabeth Cortier, Vivien Rolland, Peter Swoboda, Bénédicte Durand

**Affiliations:** 1 Université de Lyon, Lyon, F-69003, France; 2 Université Lyon 1, CNRS, UMR5534, Centre de Génétique Moléculaire et Cellulaire, Villeurbanne, F-69622, France; 3 University of Massachusetts Medical School, Department of Neurobiology, Worcester, MA 01605, USA; 4 Karolinska Institute, Department of Biosciences and Nutrition, Södertörn University College, School of Life Sciences, S-14189 Huddinge, Sweden; 5 University of Glasgow, Glasgow Biomedical Research Centre, Wellcome Centre for Molecular Parasitology and Infection and Immunity, Glasgow G12 8TA, UK

## Abstract

**Background:**

Regulatory factor X (RFX) transcription factors play a key role in ciliary assembly in nematode, *Drosophila *and mouse. Using the tremendous advantages of comparative genomics in closely related species, we identified novel genes regulated by dRFX in *Drosophila*.

**Results:**

We first demonstrate that a subset of known ciliary genes in *Caenorhabditis elegans *and *Drosophila *are regulated by dRFX and have a conserved RFX binding site (X-box) in their promoters in two highly divergent *Drosophila *species. We then designed an X-box consensus sequence and carried out a genome wide computer screen to identify novel genes under RFX control. We found 412 genes that share a conserved X-box upstream of the ATG in both species, with 83 genes presenting a more restricted consensus. We analyzed 25 of these 83 genes, 16 of which are indeed RFX target genes. Two of them have never been described as involved in ciliogenesis. In addition, reporter construct expression analysis revealed that three of the identified genes encode proteins specifically localized in ciliated endings of *Drosophila *sensory neurons.

**Conclusion:**

Our X-box search strategy led to the identification of novel RFX target genes in *Drosophila *that are involved in sensory ciliogenesis. We also established a highly valuable *Drosophila *cilia and basal body dataset. These results demonstrate the accuracy of the X-box screen and will be useful for the identification of candidate genes for human ciliopathies, as several human homologs of RFX target genes are known to be involved in diseases, such as Bardet-Biedl syndrome.

## Abstract 2

An RFX-binding site is shown to be conserved in the promoters of a subset of ciliary genes and a subsequent screen for this site in two Drosophila species identified novel RFX target genes that are involved in sensory ciliogenesis.

## Background

Eukaryotic cilia and flagella are present in many types of tissues and organisms and are important for sensory functions, cell motility, molecular transport, and several developmental processes, such as the establishment of left-right asymmetry in vertebrates [1-5]. Several human diseases are known to result from defects in ciliary assembly or function and have recently been designated as ciliopathies [5]. Cilia are well-defined structures consisting of a microtubular axoneme composed of specific proteins that are assembled dynamically in a strict stereotypical pattern (for reviews, see [6,7]). Ciliary assembly depends on intraflagellar transport (IFT) a dynamic process highly conserved in organisms ranging from the green algae *Chlamydomonas *to mammals (reviewed in [1,8,9]). Several studies in various organisms have been instrumental in the identification of genes involved in the assembly and function of the cilium. The proteomic analysis of detergent-extracted ciliary axonemes from cultured human epithelial cells identified 214 proteins [10]. More recently, a biochemical fractionation of *Chlamydomonas reinhardtii *flagella led to the identification of about 700 proteins, of which 360 had high confidence of truly being involved in flagellar composition [11]. A proteomic analysis of *Trypanosoma brucei *flagella allowed the identification of 522 proteins [12]. Two remarkable approaches took advantage of the availability of complete genome sequences to identify genes encoding ciliary and flagellar proteins. By comparing the genomes of ciliated versus non-ciliated organisms, Avidor-Reiss *et al*. [13] and Li *et al*. [14] selected 187 and 688 genes, respectively, that are specific to ciliated organisms. Stolc *et al*. [15] used microarray hybridization to analyze induction levels of all *C. reinhardtii *genes after deflagellation. They identified 220 genes that are induced at least two-fold and, therefore, are likely to be involved in the assembly or function of cilia and flagella.

Much less is known about the regulatory pathways that control the expression of ciliary components or direct the differentiation of ciliated cells. The transcription factor FoxJ1 appears to govern the differentiation of ciliated cells in vertebrates, but so far, only one gene has been shown to be directly regulated by FoxJ1 [16]. The transcription factor HNF1-β has also been shown to regulate several genes involved in ciliogenesis in the kidney [17]. Most importantly, regulatory factor X (RFX) transcription factors play a key role in regulating genes involved in ciliogenesis. RFX transcription factors are conserved in a wide range of species, including *Saccharomyces cerevisiae*, *Caenorhabditis elegans*, *Drosophila melanogaster *and mammals. They share a characteristic DNA-binding domain of the winged-helix DNA binding family and bind to an X-box motif, an imperfect inverted repeat with variable spacing between the repeats [18,19]. Whereas only one *Rfx *gene is described in yeast and *C. elegans*, two *Rfx *genes are present in the *Drosophila *genome and five in mammals [20]. Major clues on RFX functions in metazoans have been obtained from work on invertebrates. *daf-19*, the sole *Rfx *gene in *C. elegans*, is a key regulator of ciliogenesis [21]. *dRfx *in *Drosophila *is expressed in ciliated cells and is necessary for ciliated sensory neuron differentiation: all sensory neurons are present but cilia are missing at the dendritic tips [22,23]. In mouse, we have shown that RFX function in ciliogenesis is conserved. Indeed, *Rfx3 *controls the growth of mouse embryonic node cilia [24] and *Rfx3 *loss-of-function leads to hydrocephalus with differentiation defects of ciliated ependymal cells of the choroid plexus and subcommisural organ [25]. Moreover, *Rfx3 *mutant mice show insulin secretion failure and impaired glucose tolerance correlated with primary ciliary growth defects on islet cells [26]. In zebrafish, *Rfx2 *is expressed specifically in multiciliated cells of the pronephros and loss of *Rfx2 *leads to cyst formation and loss of multicilia [27]. The function of the other RFX proteins has yet to be linked to ciliogenesis. *Rfx5*, the most divergent mammalian member, regulates major histocompatibility class II gene expression and mutations in it are responsible for the bare lymphocyte syndrome [28]. *Rfx4 *has been implicated in dorsal patterning of brain development in mice and may participate in circadian rhythm regulation in humans [29-32].

Because RFX function in ciliogenesis appears conserved from *C. elegans *to mammals, X-box promoter motif sequences can guide the search for ciliary genes. Indeed, genome wide searches for genes controlled by DAF-19 in *C. elegans *have identified many genes involved in ciliogenesis [14,21,33-38]. Genomic X-box searches thus comprise a key method to identify genes involved in ciliary development. We show here that ciliogenic RFX regulatory cascades are well conserved between *D. melanogaster *and *C. elegans *and identify a first set of 14 RFX target genes. In particular, we show that all known *Drosophila *homologs of genes defective in human Bardet-Biedl syndrome (BBS), a human ciliopathy with complex phenotypes, are controlled by dRFX. Moreover, by using comparative genomic screens we show that genes under dRFX control in *D. melanogaster *share conserved X-boxes with another divergent *Drosophila *species, *D. pseudoobscura*. Applied to the whole genome of both species, our comparative approach led to the identification of at least 11 novel RFX target genes. *In vivo *reporter assay studies for three of them confirmed their involvement in ciliary structure or function in *Drosophila*, thus illustrating the accuracy of our screen. In addition, we have established a highly confident *Drosophila *cilia and basal body (DCBB) gene list and highlight several genes as novel candidates for ciliogenesis. Our data are of particular importance for further genetic and genomic studies in the field of ciliogenesis and, consequently, for identifying genes involved in human ciliopathies.

## Results

### Homologs of *C. elegans *DAF-19 target genes are regulated by dRFX in *Drosophila*

Our previous work has shown that RFX transcription factors share a common function in ciliogenesis in worm and fly [21,23]. We thus inferred that an identical set of genes would be regulated by DAF-19 in *C. elegans *and dRFX in *D. melanogaster*. Indeed, among more than 20 previously identified DAF-19 targets expressed in all ciliated sensory neurons of *C. elegans *[21,36-38], we show that a majority of the homologous genes in fly are down regulated in *dRfx *mutants (Table 1). Regulation of gene expression was tested by real-time PCR based on RNA extracted from 40-hour old pupae thoraxes and legs. At this stage, dendrites and cilia have just differentiated. Moreover, the levels of expression of ciliary genes *osm-6 *and *nompB*, relative to the housekeeping gene *TBP *(*TATA Binding Protein*) or the pan-neural gene *elav *during pupae development, is at a maximum starting at 40 hours after puparium formation (data not shown). As shown in Table 1, 14 of 19 DAF-19 regulated genes for which a homologous gene can be found in *Drosophila *are also regulated by dRFX. Only one gene (*CG5359/D1009.5/xbx-2/dylt-2*) regulated by DAF-19 in all ciliated sensory neurons in *C. elegans *does not seem to be under dRFX regulation in *Drosophila*. Among all the *C. elegans *genes expressed and regulated by DAF-19 in a subset of ciliated sensory neurons, only *CG9398/tulp *appears to be under dRFX control in *Drosophila*. All the others, such as *oseg3*, *NudC *or *amo*, do not appear to be regulated by dRFX in our assay conditions. However, we cannot exclude that these genes are under dRFX regulation in a small subset of ciliated sensory neurons and, thus, that variations of their expression cannot be detected by real time RT-PCR of RNA preparations of pupae thoraxes and legs. Remarkably, genes that are involved in BBS and conserved in both organisms are regulated by RFX proteins. We quantified the expression of *CG13232/BBS4 *in *Drosophila*, the only *BBS *gene that is not found in the *C. elegans *genome, and show that it is also down regulated 17-fold in a *dRfx *deficient background. Most of the other genes regulated by dRFX are involved in IFT. This transport is led by two types of molecular motors, anterograde kinesins and retrograde dyneins, that carry particles that can be biochemically fractionated as A and B complexes [1]. dRFX regulates genes encoding B complex components, but not A complex components.

**Table 1:** RFX target genes in *C. elegans *and *D. melanogaster *and in compartmentalized ciliogenesis

*D. melanogaster *gene ID (name)	Homologs in vertebrates or *Chlamydomonas*	Fold variation	Ciliary type [13]	*C. elegans *gene ID (name)	*DAF-19 *control in *C. elegans*
**Downregulated >2 fold**					
CG1126	*BBS5*	95.2*	Cp	R01H10.6 (*bbs-5*)	All [14]
CG3769	*D2LIC*/*LIC3*	63*	-	F02D8.3 (*xbx-1*)	All [21,35]
CG4525	Novel	223.6*	Cp	C27H5.7 (*dyf-13*)	All [37]
CG8853	*IFT55/hippi*	21.8*	Cp	F59C6.7 (*che-13*)	All [33]
CG9333 (*oseg5*)	*WDR56*	4.4*	Cp	F38G1.1 (*che-2*)	All [21]
CG9595 (*osm-6*)	*NDG5*	22.2*	Cp	R31.3 (*osm-6*)	All [21]
CG12548 (*nompB*)	*TG737*	12.7 [92]*	Cp	Y41g9a.1 (*osm-5*)	All [34]
CG13691 (*BBS8*)	*BBS8*	2.7*	Cp	T25F10.5 (*bbs-8*)	All [36]
CG13809 (*oseg2*)	*IFT172/wim*	9.7*in vivo	Cp	T27B1.1 (*osm-1*)	All [21]
CG14825 (*BBS1*)	*BBS1*	211*	Cp	Y105E8A.5 (*bbs-1*)	All [36,37]
CG15666	*BBS9*	20*	-	C48B6.8	All [37]
CG17599	*QILIN*	29.5*	Cp	C04C3.5 (*dyf-3*)	All [93]
CG9398 (*Tulp*)	*Tubby*	3.2^‡^	-	F10B5.4 (*tub-1*)	Subset [36]
CG3259	*Traf3ip1/MIP-T3*	*In vivo*	Cp	C02H7.1	ND
CG5142	Novel	16.4*	Cp	F54C1.5a (*dyf-1*)	ND
CG7735	*BBS3*	40.9*	Cp	C38D4.8 (*arl-6*)	ND
CG9227 (*tectonic*)	*tectonic*	*In vivo*	Cp	Y38F2AL.2	ND
CG14367	Novel	10.2^†^	Cp	Y108G3AL.3	ND
CG14870	B9 domain	3.1*	Cp	K03E6.4	ND
CG15161	*IFT46*	207.1*	Cp	F46F6.4 (*dyf-6*)	ND
CG18631	Novel	14.2*	Cp	K07G5.3	ND
CG13232 (*BBS4*)	*BBS4*	17*	-	-	-
					
**Downregulated <2-fold**					
CG30441	*IFT20*	2^‡^	Cp	Y110A7A.20	All [37]
CG1399-PB	*LRRC16*	1.8^†^	Cp	K07G5.1	ND
CG11048	*Rib74*	1.5^†^	Cp	Y49A10A.1	ND
CG13178	Novel	1.7^†^	Cp	R10F2.5	ND
					
**Overexpressed**					
CG2006	*Dtwd1*	0.5*	Cp	Y53C12A.3	
					
**Invariant in *Drosophila***					
CG5359	*Tctex*	1		D1009.5 (*xbx-2/dylt-2*)	All [36]
CG11237 (*oseg6*)	*wdr19*	1	Cp	ZK520.3 (*dyf-2*)	Subset [94]
CG6504 (*amo*)	*PKD2*	0.9		Y73F8A.1 (*pkd-2*)	Subset [95]
CG9710 (*nudC*)	*NudC*	0.7		F53A2.4 (*nud-1*)	Subset [36]
CG11838 (*oseg3*)	*IFT140*	1.4	Cp	C27A7.4 (*che-11*)	Subset [36]
CG2069 (*oseg4*)	*WDR35*	1	Cp	C54G7.4 (*ifta-1*)	ND
CG6560	*arl3*	1	Cp	F19H8.3 (*arl-3*)	ND
CG7161 (*oseg1*)	*IFT122*	1.4 *in vivo*	Cp	F23B2.4 (*daf-10/osm-4*)	ND
CG10642 (*Klp64D*)	*KIF3A*	1.1	Cp	Y50D7A.6 (*klp-20*)	ND
CG11755	Novel	1.1	Cp	D2089.3	ND
CG11759 (*Kap3*)	*KIFAP3*	1	Cp	F08F8.3 (*kap-1*)	ND
CG5195	L domain like	1.2	Cp	-	-
CG31249	Novel	0.9	Cp	-	-
CG32392	*rshl2*	1.1	Cp	-	-
					
**Not determined in *Drosophila***					
CG3798 (*Nmda1*)		ND		F40F9.1 (*xbx-6*)	Subset [36]
CG9310 (*Hnf4*)	*HNF4A/G*	ND		T19A5.4 (*nhr-44*)	Subset [36]
CG17228 (*pros*)	*PROX1/2*	ND		K12H4.1 (*ceh-26*)	Subset [95]
					
**Not conserved in *Drosophila***					
-		-		Y75B8A.12 (*osm-12*/*bbs-7*)	All [36]
-		-		F20D12.3 (*bbs-2*)	All [36]
-		-		M28.7 (*nph-1*)	All [96]
-		-		R13H4.1 (*nph-4*)	All [96]
-		-		C47E8.6	All [37]
-		-		ZK328.7a	All [37]
-		-		Y102E9.1 (*odr-4*)	Subset [36]
-		-		C23H5.3 (*xbx-4*)	Subset [36]
-		-		M04D8.6 (*xbx-3*)	Subset [36]
-		-		T24A11.2 (*xbx-5*)	Subset [36]
-		-		R148.1 (*xbx-7*)	Subset [36]
-		-		D2005.2 (*nlp-8*)	Subset [95]

*p value < 0.001; ^†^0.001 <*p *value < 0.05; ^‡^0.05 <*p *value < 0.08. Dashes indicate not applicable. Cp, compartmentalized ciliogenesis; ND, not determined.

### Genes specific to compartmentalized ciliogenesis are regulated by dRFX in *Drosophila*

Interestingly, most of the genes regulated by dRFX also fall in the list of genes for compartmentalized ciliogenesis (Cp ciliary type, Table 1) defined by the work of Avidor-Reiss *et al*. (Table 1) [13]. This group of genes is found only in genomes of species showing compartmentalized cilia biogenesis, but neither in the genomes of non-ciliated organisms nor in *Plasmodium falciparum*, which uses cytosolic cilia biogenesis. We thus tested the expression of almost all the genes described in the Cp category in control and *dRfx *deficient *Drosophila*. Among the 34 Cp ciliary genes tested by real-time PCR, 18 were down regulated more than 2-fold in a *dRfx *mutant background, 4 were significantly reduced between 1.5- and 2-fold and one was significantly over expressed. Eleven genes did not show significant expression variations between control and mutant background (Table 1).

**Figure 1:**
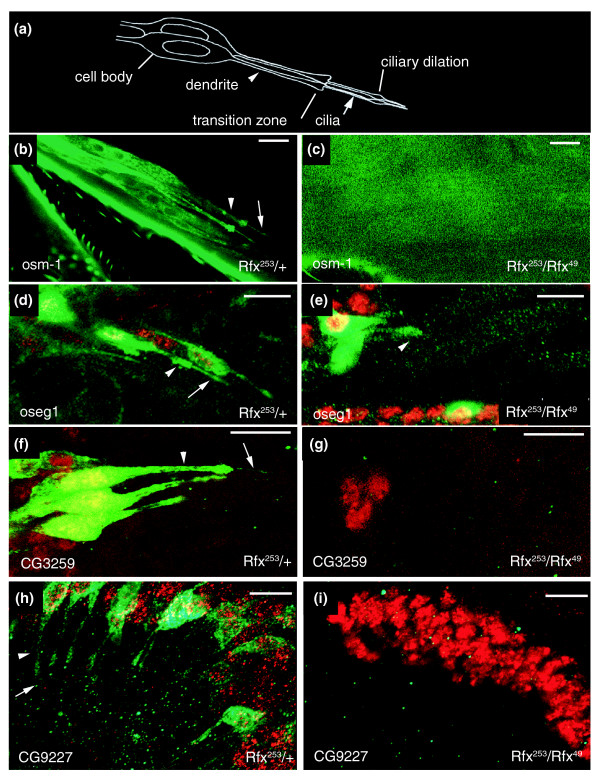
*In vivo *observations of reporter constructs in control or *dRfx*-deficient *Drosophila*. **(a) **Schematic of two typical chordotonal organs of the *Drosophila *leg or antenna. The different segments of the dendrite and of the ciliated ending are shown. Sensory neurons have a single cilium (arrow) extending from their dendrite (arrowhead). **(b) **Live confocal image of GFP driven expression of *osm-1 *transgene in a control femur. **(c) **GFP expression is totally shut down in a *dRfx *mutant background. **(d-i) **Confocal imaging of chordotonal neurons labeled with anti-ELAV (red) and anti-GFP (green). *oseg1-GFP *expression in (d) control flies and (e) a *dRfx *mutant background. Note that *oseg1-GFP *expression is not affected in the mutant background. *CG3259-GFP *expression in (f) control flies and (g) *dRfx *mutant flies. Reporter construct expression is totally shut down in the mutant background. Johnston's organs from antennae of adult flies carrying *CG9227-GFP *transgenes in (h) control and (i) *dRfx *mutant pupae. Note the absence of expression in the mutant background. Scale bar = 10 μm.

In order to demonstrate the accuracy of our quantification procedure, we performed *in vivo *observations of reporter constructs of some of the genes in wild-type and *dRfx *deficient backgrounds (Figure 1). As previously published, sensory neuron ciliary endings are missing in a *dRfx *deficient background [23]. As observed in the cell body or remaining dendrite, the expression of *osm-1 *is totally shut down in the *dRfx *deficient background, whereas the expression of *oseg1 *is not affected (Figure 1), in agreement with real-time RT-PCR results. Interestingly, *CG3259 *and *CG9227 *cDNAs were hardly detectable by real-time PCR and, thus, difficult to quantify. However, *in vivo *observations of reporter constructs in wild-type and *dRfx *mutant backgrounds show a complete absence of expression of these two genes in the mutant background (Figure 1).

In summary, we show that RFX target genes are mainly conserved between *C. elegans *and *D. melanogaster*. Our functional comparative approach between both organisms combined with the work of Avidor-Reiss *et al*. in *Drosophila *allowed us to identify 27 genes that are regulated by dRFX in *Drosophila*. A majority of them are shown to be involved in ciliogenesis.

### X-box conservation between *D. melanogaster *and *D. pseudoobscura*

As previously described [13,14,21,36-39], the X-box promoter motif has been used successfully to screen for genes involved in ciliogenesis. As shown above, this first set of X-box gene data in *Drosophila *is thus a key to better understand the link between X-box sequences and dRFX transcriptional control in *Drosophila*. We looked for X-boxes in the promoters of dRFX target genes. We searched for X-boxes up to 3 kb upstream of the ATG for each of them, with the most degenerated X-box consensus deduced to date from known RFX protein binding sites (RYYNYY N1-3 RRNRAC). We could identify several X-boxes for each gene (Table 2, columns 2 and 3). However, known negative control genes also presented X-boxes at the same frequency and no particular constraint on the consensus seemed to correlate with one set of genes. Therefore, the presence for one gene of an X-box upstream of its ATG is not predictive of dRFX-dependent expression. We thus turned to the *D. pseudoobscura *genome. The two *Drosophila *species' most recent common ancestor occurred 40-60 million years ago. The average identity of coding sequence between *D. melanogaster *and *D. pseudoobscura *at the nucleotide level is 70% for the first and second bases of codons, and 49% for the wobble base. Intron sequences are 40% identical, untranslated regions 45-50%, and DNA protein binding sites extracted from the literature have been estimated to an average of 63% [40]. Moreover, detailed comparison of both *Drosophila *genomes showed that 50-70% of known DNA binding sites reside in conserved sequence blocks in the genomes, called conserved regulatory elements (CREs), whereas the overall conservation of the cis-regulatory regions is low [41-43].

**Table 2:** X-box comparisons in promoters of dRFX regulated genes, between *Drosophila melanogaster *and *Drosophila pseudoobscura*

***D. melanogaster***			***D. pseudoobscura***				***D. melanogaster***		***D. pseudoobscura***			
	No. of X-box			No. of X-box								

	Strand			Strand							Conserved blocks around X-box^†^	

Gene ID	+	-	Gene ID	+	-	No. of conserved X-box*	X-box	Dist.	X-box	Dist.	100 bp	25 bp

**Genes down regulated in *dRfx *mutant**												
CG1126-PA	2	4	GA10872-PA	2	1	1^‡^	GTTGCC T AGCAAC	66	GTTGCC T AGCAAC	91	-	+
CG3259-PA	4	4	GA17011-PA	2	2	1^‡^	GTTGCC AG GACAAC	81	GTTGTC AG GACAAC	96	-	+
CG3769-PA	2	3	GA17674-PA	4	3	1^‡^	GTTGCT AGT AGCAAC	71	GTTGCC AG GACAAC	56	-	+
CG4525-PA	2	3	GA18233-PA	1	1	1^‡^	GTTGCC A AGCGAC	134	GTTGCC A AGCGAC	239	+	+
CG8853-PA	2	4	GA21369-PA	1	5	1^‡^	GTTACC TT GGCGAC	87	GTTACC AT GGAAAC	110	-	+
CG9227-PA	5	1	GA21627-PA	3	1	1^‡^	GTTACT TT GACAAC	119	GTTGCC AG AGCAAC	146	+	+
CG9595-PA	3	2	GA21901-PA	3	2	1^‡ ^+ 2	GTTGCC G GGCAAC	126	GTTGTC CG GGCAAC	141	+	+
							ATTTTT GTT AGCAAC	264	ACTTTT GC AAAAAC	699	-	+
							GCTGTT ACA AGAGAC	2,969	GCTGCT GCA GGAAAC	2,671	NA	NA
CG12548-PA	7	2	GA11690-PA	2	4	2	ATCACC AA GGCAAC	2,335	ATCACC TT GGAAAC	868	-	-
							GCCTTT C GGAGAC	2,833	GCCGCT T GATGAC	2,638	-	-
CG13178-PA	4	2	GA12098-PA	5	4	1	GCCGTT AGC AAGAAC	2,551	GCCACC AGG AAAAAC	2,106	NA	NA
CG13809-PA	1	2	GA12544-PA	2	2	1^‡^	GTTGCC AC AACAAC	123	GTTGCC AC AACAAC	103	+	+
CG15161-PA	2	1	GA13541-PA	4	2	2^‡^	GTTGTC AG GACGAC	321	GTTGTC AA GACAAC	311	-	-
							GTTGTC AG GACGAC	321	GTTTTT GCA GGCAAC	391	-	-
CG30441-PA	1	3	GA15848-PA	3	1	1^‡^	GTTGTC AAT AGCAAC	60	GTTGTC TGT GACAAC	122	-	-
CG17599-PA	1	5	GA14573-PA	1	1	1^‡^	GTTACC T AGCAAC	141	GTTGCC T GGCAAC	206	-	-
CG18631-PA	2	2	GA15024-PA	6	1	1^‡^	GTTGCC CAT GGCAAC	2,731	GTTGCC GTT AGCAAC	2,633	-	+
CG15666-PA	3	3	GA13881-PA	2	3	1^‡^	GTTGCC AA GGCAAC	88	GTGGCC AT GGCAAC^§^	5	-	-
CG14870-PA	1	1	GA13310-PA	1	1	1^‡^	GTCTCC CG GGCAAC	-22	GTATCC TG GGCAAC^§^	-6	+	+
CG1399-PB	3	3	GA12678-PA	3	0	0						
CG5142-PA	3	2	GA18687-PA	2	1	0						
CG9333-PA	4	1	GA21709-PA	2	1	0						
CG9398-PA	2	2	GA21760-PA	3	3	0						
CG11048-PA	1	0	GA10726-PA	5	4	0						
CG13691-PA	1	1	GA12462-PA	1	1	0						
CG14367-PA	0	3	GA12937-PA	3	3	0						
												
**Invariantly expressed genes in *dRfx *mutant**												
CG5195-PA	0	4	GA18727-PA	3	4	0						
CG5359-PA	4	4	GA17011-PA	2	1	1^‡^	GTTATT CGT GGTGAC	141	GTTGTT G AGGAAC	51	-	-
CG6504-PA	3	0	GA19567-PA	6	4	0						
CG6560-PA	3	0	GA19685-PA	0	2	0						
CG7161-PA	2	5	GA20145-PA	0	3	0						
CG9710-PA	2	0	GA21982-PA	1	0	0						
CG10642-PA	4	2	GA10463-PA	1	3	1^‡^	GTCGTT TAA GGAAAC	1,041	GTTGTC ATG AGTAAC	1,226	-	+
CG11755-PA	1	5	GA11176-PA	2	1	1	ACCGCC CAG AAGAAC	2,418	ACTACC GT GAAAAC	1,576	NA	NA
CG11237-PA	3	2	GA10856-PA	1	2	0						
CG11759-PA	1	3	GA11178-PA	3	2	0						
CG11838-PA	3	0	GA11225-PA	3	4	0						
CG31249-PA	1	1	GA16123-PA	1	1	0						
CG32392-PA	4	2	GA16867-PA	3	3	0						

*RYYNYYN[1-3]RRNRAC. Dist., distance upstream of the ATG. ^†^The percentage of identities of either 100 bp or 25 bp sequence blocks around the X-box was estimated by Vista comparison as positive (+) if identities are above 50%, or negative (-) if below 50%. ^‡^Palindrome. ^§^The X-box fits the more degenerated consensusRYNNYYN[1-3]RRNRAC. NA, not applicable because of nearby coding sequence environment or gap in the sequences.

We thus looked for *D. pseudoobscura *homologs of either dRFX positively regulated or invariant genes and for X-boxes up to 3 kb upstream of the ATG. Interestingly, 70% of conserved dRFX target genes present a conserved X-box in both species (Table 2), whereas only 23% of negative control genes present the same characteristic. Even more precisely, while the sequence and the location of X-boxes for dRFX target genes are conserved, this is not the case for negative control genes. Interestingly, palindromic X-boxes are significantly over-represented compared to non-palindromic X-box sequences in dRFX regulated genes in the two species.

**Figure 2:**
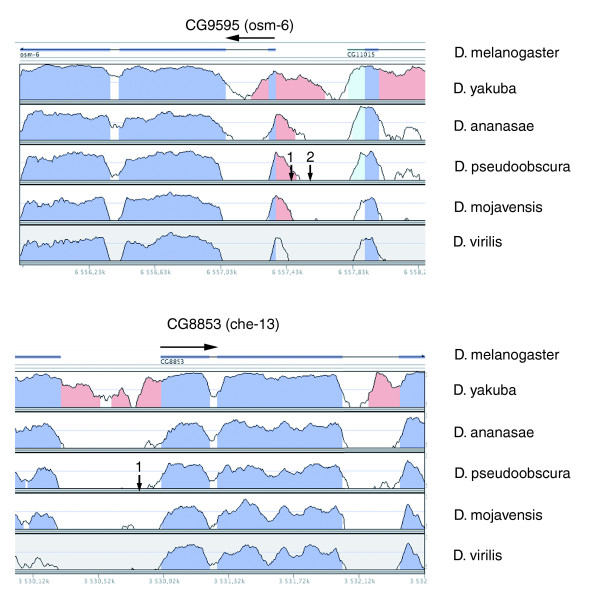
Promoter comparisons between *Drosophila *species. Sequence identities (from 50-100%) between different *Drosophila *species ranging from *D. melanogaster *to the most distant *D. virilis *as calculated and presented in the VISTA interface [91] for two *dRfx *target genes, *CG9595 *(*osm-6/NDG5*) and *CG8853 *(*IFT55/che-13/Hippi*). Coding sequences are depicted in dark blue, untranslated regions are in light blue and other conserved regions in pink. Gene orientation is shown by a horizontal arrow. The location of conserved X-boxes for each gene is indicated by numbered vertical arrows. Note that one conserved X-box for *osm-6 *is in a conserved block of sequence, while others (*osm-6 *and *che-13*) are not.

We also looked for overall sequence conservation around the selected X-boxes by Vista promoter sequence comparison between the two *Drosophila *species. The percentage of identities was quantified either on 100 bp or 25 bp windows surrounding the X-boxes (Figure 2, Table 2) and block conservation was considered positive if identities were over 50%. As shown in Table 2, sequences around the X-boxes are generally not well conserved. Two representative examples are depicted in Figure 2. For the *CG9595/osm-6 *gene, one of the two conserved X-boxes falls into an overall conserved 100 bp block, whereas the other one does not. For *CG8853*/*che-13*, the X-box falls into a poorly conserved region. These results are in agreement with previously published data showing that sequence block conservation alone cannot discriminate regulatory regions, but that binding site clusters present in multiple species more likely discriminate active and inactive clusters [43].

### Screening *Drosophila *species' genomes for dRFX regulated genes

The presence of a conserved X-box upstream of genes in both *D. melanogaster *and *D. pseudoobscura *is thus a good prognostic factor to predict novel dRFX target genes. We thus screened the genome of both *Drosophila *species for the presence of X-boxes. We searched for all possible matches to a defined motif sequence using a Perl based algorithm [36]. The most degenerated consensus RYYNYY N1-3 RRNRAC found 50,000 hits throughout the entire genome of *D. melanogaster *and, therefore, could not be used within our experimental framework. We selected five different more restricted consensus motifs that cover X-boxes of the entire set of known target genes at the time (see Materials and methods). Four (RYYVYY N1-3 RRHRAC, GYTNYY N1-3 RRNRAC, GYTDYY N1-3 RRNRAC, GYTRYY N1-3 RRHRAC) were searched in a 1 kb window upstream of the ATG, and the less degenerated one, RTNRCC N1-3 RGYAAC, in a 3 kb window.

Under these conditions, 4,726 non-redundant genes in *D. melanogaster *and 3,848 in *D. pseudoobscura *with an X-box upstream of the start codon were selected. Based on a best hit reciprocal search between the two coding sequence (CDS) lists, we identified 1,462 homologous genes having an X-box in their 5' region in both species. This first set of 1,462 genes was further restricted by selecting only genes that share an X-box with no more than 4 bases different (out of the 12 nucleotides recognized by the protein on either side of the spacer) between each species and in a conserved position upstream of the ATG (500 bp difference at most). The list was thus restricted to a subset of 412 genes (Additional data file 1). An even more restricted subset of genes was selected using the X-box motif GYTRYY N1-3 RRHRAC, which was found upstream of most known target RFX genes at the beginning of this work, leading to a list of 83 genes (Table 3). Indeed, among the identified dRFX target genes for which a conserved X box was found in both *Drosophila *species (Table 2), the highest percentage of target genes (50%, 8 out of 16) was found in this list of 83 genes. The remaining 50% of known RFX target genes (Table 2) were not selected by the X-box screen and thus represent false negatives (see Discussion for a comprehensive analysis).

**Table 3:** Eighty-three genes selected for a conserved X-box between *D. melanogaster *and *D. pseudoobscura*

					Source [reference]														
CG# release 3.2.1	Rfx target fold reduction	Flybase gene name	Known protein function	Annotated predictive function	[12]	[11]	[14]	[13]	[10]	[15]	[36]	[37]	[45]	[44]	[59]	DCBB	Ciliome database	Ciliary proteome database	Human homologs

1126	95	*CG1126*	BBS5				ν	ν			ν	ν				ν	ν	ν	NP_689597
2321	-	*CG2321*	-																NP_060350.1
2691	-	*CG2691*	-																NP_055994.1
3259	*iv*	*CG3259*	MIP_T3			ν	ν	ν		ν		ν			ν	ν	ν	ν	NP_056465
3344	-	*CG3344*		Serine carboxypeptidase															NP_067639.1
3603	-	*CG3603*		Rhodopsin-like receptor													ν		NP_055049.1
3723	3	*Dhc93AB*	DNAH9	ATPase activity, coupled	ν	ν	ν		ν							ν	ν	ν	NP_001363
3769	63	*CG3769*	xbx1/D2LIC			ν	ν				ν					ν	ν	ν	NP_057092
4135	-	*beat-IIb*		IgSF protein, axon guidance															1 to many
4314	-	*st*	scarlet	ATPase															1 to many
4536	6	*iav*	TRPV4														ν		NP_067638.3
4857	-	*CG4857*	-																-
4984	-	*CG4984*	-																NP_114102.2
5148	-	*CG5148*	-																-
5155	Neg	*CG5155*	-	Binding					ν							ν	ν	ν	NP_060546
5599	-	*CG5599*		Acetyltransferase													ν	ν	NP_001909
6054	Neg	*Su*(*fu*)	Su(fu)	Kinase inhibitor															NP_057253.2
6121	Neg	*Tip60*		Histone acetyltransferase													ν		NP_874369.1
6129	6/*iv*	*CG6129*	rootletin						ν					ν		ν		ν	NP_055490
6405	-	*CG6405*	-																XP_936274.2
6665	-	*CG6665*	-																NP_056264.1
6696	-	*CG6696*		Meprin A/proteolysis														ν	NP_005916
7104	-	*Spz3*																	-
7669	-	*CG7669*							ν							ν			NP_002815.3
7754	-	*iotaTry*		Trypsin															1 to many
8344	-	*RpIII128*		RNA polIIIb													ν		NP_060552.3
8362	Neg	*nmdynD7*		Nucleoside diphosphate kinase	ν	ν	ν			ν			ν	ν	ν	ν	ν	ν	NP_037462
8853	23	*CG8853*	IFT55/Hippi			ν	ν	ν	ν	ν	ν	ν				ν	ν	ν	NP_060480
8971	-	*fh*	frataxin like																NP_000135.2
9035	-	*Tapdelta*		Signal sequence binding															NP_006271.1
9227	*iv*	*tectonic*	Tectonic				ν	ν			ν	ν				ν	ν	ν	NP_085055
9298	-	*CG9298*		Synbindin															NP_057230.1
9363	-	*CG9363*		Glutathione transferase zeta															NP_665877.1
9467	-	*CG9467*		V-gated K channel complex															NP_057205.2
9689	-	*CG9689*	-																-
9764	-	*yrt*	yurt	Cytoskeletal protein binding															NP_060894.2
10035	-	*CG10035*	-																-
10068	-	*CG10068*	-																NP_714913.1
10143	-	*Adgf-E*		Adenosine deaminase															NP_803124.1
10601	-	*mirr*		Protein binding														ν	NP_077311
10859	-	*CG10859*	DIC	Motor activity															NP_075462
11015	-	*CG11015*	COX5B	Cytochrome-c oxidase													ν		NP_001853
11164	-	*CG11164*	-																NP_078846.1
11356	-	*CG11356*	ARL13B	GTP binding															NP_878899
11426	-	*CG11426*		Lipid metabolism															1 to many
11438	-	*CG11438*		G protein coupled pathway															NP_795714.1
11529	-	*CG11529*		Chymotrypsin activity															NP_005037.1
11983	-	*CG11983*	-																-
12020	-	*CG12020*	-	Heat shock protein binding		ν				ν						ν			NP_705842.2
13125	5	*CG13125*		LRR	ν	ν	ν								ν	ν	ν	ν	NP_112584
13202	-	*CG13202*	-																-
13216	-	*CG13216*	-																-
13251	-	*CG13251*	-																NP_001012524.1
13271	-	*Ugt36Bb*		Glucuronosyltransferase															1 to many
13415	2	*Cby*	Chibby																NP_056188.1
13432	Neg	*l*(*2*)*05510*	-																-
13809	172/*iv*	*osm-1*	IFT172/wim			ν	ν	ν	ν		ν	ν				ν	ν	ν	NP_056477
14079	-	*CG14079*	-																NP_061946.1
14127	Neg	*CG14127*	-						ν							ν	ν	ν	NP_659482
14313	-	*CG14313*	-																-
14430	-	*CG14430*	-																-
14661	-	*CG14661*	-																-
14791	Neg	*Rab27*		GTP binding				ν								ν	ν	ν	NP_004571
15148	124	*btv*	DNCH2	ATPase activity, coupled		ν	ν		ν							ν	ν	ν	NP_001073932
15161	207	*CG15161*	IFT46/dyf-6			ν	ν	ν	ν	ν						ν	ν	ν	NP_064538
15564	-	*CG15564*	-																-
15878	-	*CG15878*	-																-
17150	2	*CG17150*	DNAH3	Motor activity		ν	ν									ν			NP_060009
17259	-	*CG17259*		Seryl trna synthetase													ν		NP_006504.2
17284	-	*Obp93a*		Odorant binding															-
17785	Neg	*Golgin84*																	NP_005104.2
18432	-	*CG18432*																	NP_001073991.1
18584	-	*CG18584*	UNC84																NP_056189.1
18869	-	*CG18869*		UDP glycosyl transferase															-
30021	-	*skf*		Guanylate kinase															NP_775767.2
30441	2.8	*CG30441*	IFT20			ν		ν		ν	ν					ν	ν		NP_777547.1
31036	4/*iv*	*CG31036*	-																-
31127	-	*Wsck*		Protein-tyrosine kinase															NP_005223.3
31257	-	*CG31257*	-																-
31321	-	*CG31321*	-																NP_542400.2
31640	-	*CG31640*	-	Protein-tyrosine kinase															NP_001014796.1
31824	-	*CG31824*	-	Trypsin															-
33038	Neg	*CG8433*	exostosin2																NP_056499.2

Listing established with the restricted GYTRYYN{1-3}RRHRAC X-box consensus. Neg, invariant expression in *dRfx *deficient background compared to wt; *iv*, *in vivo *confirmation of reporter construct down regulation in *dRfx *deficient background compared to wild type. The presence of a gene in other published studies is noted as ν.

### X-box genes and ciliogenesis

In order to check for enrichment of genes involved in ciliogenesis, we compared our three X-box gene lists to previously published lists of genes potentially involved in cilium or centrosome composition. We first identified the *Drosophila *homologs for the full set of previously published genes from various organisms from several studies. These include comparative genomic studies of species that have cilia versus species that do not and proteomic analyses of human cilia and centrosome, *Chlamydomonas *flagellar or basal body and *Trypanosoma brucei *proteomes [10-14,44,45]. This set also includes recent genome-wide transcriptional analysis of gene expression during flagellar regeneration in *Chlamydomonas *or identified by SAGE analysis of ciliated neurons combined with X-box searches in *C. elegans *[15,36,37]. The full set of *Drosophila *homologs that we found for all studies combined is listed as the DCBB gene set (Additional data file 2).

Interestingly, comparing our set of 1,462 *Drosophila *X-box candidate genes with the DCBB dataset shows that our list is slightly enriched in DCBB genes. Whereas 5% of the *D. melanogaster *genome is in the DCBB dataset, our 412 and the 83 X-box gene candidate datasets appear to be highly enriched in DCBB genes (11% and 22%, respectively), suggesting that the X-box conservation is a good marker for genes potentially involved in ciliogenesis (Table 4).

**Table 4:** Comparisons of *Drosophila *X-box candidate genes with the *Drosophila *cilia and basal body genes

	Total no. of genes	X-box gene candidates		
		Total	Conserved X-box	Restricted consensus

In *D. melanogaster*	14,500	4,726		
In *D. pseudoobscura*	10,000	3,848		
**No. of homologous genes in both species**	**9,815**	**1,462**	**412**	**83**
**No. of DCBB genes in *D. melanogaster***	**815**	129 (15.8%*)	**47**	**19**
Stolc *et al*. [15]	88	28 (31.9%^†^)	14	6
Ostrowski *et al*. [10]	126	28 (22.2%^†^)	13	9
Pazour *et al*. [11]	192	41 (21.3%^†^)	18	12
Avidor-Reiss *et al*. [13]	188	38 (20.3%^†^)	14	8
Li *et al*. [14]	260	50 (19.2%^†^)	25	12
Blacque *et al*. [37]	50	9 (18.0%^†^)	6	5
Broadhead *et al*. [12]	69	15 (22.1%^†^)	5	3
Efimenko *et al*. [36]	117	20 (16.7%^†^)	8	6
Keller *et al*. [45]	51	9 (17.7%^†^)	3	1
Andersen *et al*. [44]	56	8 (14.3%^†^)	2	2
**Percent X-box genes in the DCBB dataset**		**8.82%**	**11.41%**	**22.89%**

* Percent of the 815 different DCBB genes. ^†^Percent of total genes for each study present in the X-box list. See Additional data file 2 for the DCBB dataset.

**Figure 3:**
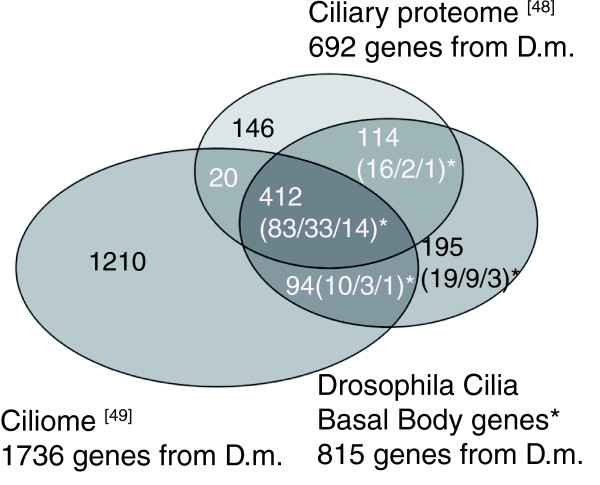
Comparison of the DCBB set of genes with the Ciliary proteome and Ciliome databases. Venn diagram presenting the overlaps between the three datasets: the cilia proteome [46,48]; the ciliome [47,49], and the DCBB (Additional data file 2). Asterisks indicate this study. Note that only 412 common genes are found in the three datasets. The number of genes also found in the 1,462, 412 or 83 X-box gene lists (Table 4), respectively, are noted in parentheses. The numbers of genes selected in the different studies to construct each dataset are given in Additional data file 3.

The full set of genes with a putative function in ciliogenesis has also been summarized in parallel in two independent databases called the Ciliary proteome and Ciliome databases [46-49]. Surprisingly, when we compared the two published databases with the DCBB dataset that we established for *Drosophila *using similar comparative methods (see Materials and methods and Additional data file 2), we observed large discrepancies between all three datasets (illustrated in Figure 3 and Additional data file 3). There are some differences between the three studies with regard to the initial published sets of genes that were included in the database. The major difference resides in which data are included from the work of Blacque *et al*. [37]. The Ciliome database [47] includes the complete SAGE dataset from Table S1 in [37], whereas our DCBB dataset includes only data from Table 1 from Blacque *et al*. (2005), which contains part of the SAGE data combined with an X-box search. The ciliary proteome database [46] includes data from Table S4 of the Blacque *et al*. study [37], which reports the list of putative X-box genes in the nematode. These differences could account for the high number of genes exclusively represented in the Ciliome database [47] but cannot account for all the discrepancies between our DCBB dataset and the Ciliary proteome database [46] (Additional data file 3). Very likely, the differences observed between all three studies illustrate the problems inherent in automatically processing published tables and gene lists that are then used to compile homologous genes from several different organisms. Another major explanation for the observed discrepancies resides in the order BLAST searches were performed to create each database. For example, the Ciliary proteome database [46] was obtained by looking first for human homologs for each study, and then for the *Drosophila *ones (unless *Drosophila *was the starting study). In our DCBB dataset, we have looked for *Drosophila *homologs, which were then compared to other datasets. Hence, genes that do not have an ortholog in *Drosophila *or in human are lost in the respective studies.

However, we show that our lists of 412 and 83 X-box genes are enriched in genes involved in ciliogenesis, whatever database is considered (Table 3, Additional data file 1). Thus, our genome wide X-box consensus motif search allowed the establishment of promising sets of candidate genes for ciliogenesis studies.

### Functional analysis of identified X-box genes

We performed functional expression studies to determine whether or not some of the 83 X-box genes (Table 3) are indeed under dRFX control and if they are involved in ciliogenesis. Twenty-five genes were tested by real time RT-PCR to compare their levels of expression in wild-type versus *dRfx *deficient fly samples. Interestingly, 16 are under dRFX control (Table 3, fold variation indicated in column 2). Among them, 11 have not yet been described as RFX targets in any biological system and two of them have no assigned function as of yet. Nine genes were not found to be under dRFX control (Table 3, noted as 'Neg' in column 2). Among 19 genes also represented in the DCBB dataset (Table 3, Additional data file 2), 17 were tested by real time PCR. Fourteen are indeed regulated by dRFX and only three do not appear to be regulated by it. The two remaining genes were not amplified by real time RT-PCR and, thus, could not be analyzed by this approach. Interestingly, among six genes that were not found in any ciliary database and whose expression was quantified by real-time PCR, two (*CG13415/Cby*, *CG31036*) were down-regulated in *dRfx *mutants. Thus, a high proportion of the genes on the list of 83 X-box genes are indeed dRFX target genes. The 58 remaining genes from this list that have not yet been analyzed are thus promising candidates. Our whole genome screen led to the identification of novel dRFX target genes.

Among the 11 novel dRFX target genes that we identified in this screen and that have never been described as RFX target genes in any organism, 9 do have a described or highly predictive function in ciliogenesis in other organisms. For example, *CG15161 *encodes the homolog of the IFT46 subunit in *Chlamydomonas *[50] and the *dyf-6 *ciliary gene in *C. elegans *[51]. *CG15148/btv*, *CG3723 *and *CG17150 *encode different dynein subunits. *beethoven *(*btv*) mutants show defects in sensory cilia in *Drosophila *[52], whereas no functional studies are available for either *CG3723 *and *CG17150 *or their orthologs in any biological system. *CG6129 *is the only *Drosophila *member of the rootletin family of proteins. In mammals, rootletin is necessary for retinal cilia stability and centrosome cohesion in mammalian cells [53-56]. *CG4536/osm-9 *encodes a vanilloid receptor of the transient receptor potential (TRP) family of ion channels. *osm-9 *is involved in sensory cilia function in *Drosophila *and *C. elegans*, and in mammals, TRPV4 plays a crucial role in ciliary activity [57]. *CG9227/Tectonic *has been described as being involved in *Shh *signaling in mouse [58]. It has been isolated by comparative genomics as a candidate for ciliogenesis and shown to be specific to ciliated cells in *Drosophila *[13]. *CG13125 *has recently been shown to be specific to species with motile cilia and its homolog, *TbCMF46*, is necessary for flagellar motility in *T. brucei *[59]. *CG3259 *encodes the MIP-T3 protein that associates with the tumor necrosis factor receptor in human cells. It is also an inhibitor of the IL13 signaling pathway that is known to repress ciliary differentiation of human epithelial cells *in vitro *[60-62]. It is expressed in ciliated sensory cells in *Drosophila *[13]. Thus, the gene *CG3259 *may have a direct function in ciliogenesis, which functional studies in *Drosophila *will allow to be deciphered.

Interestingly, two novel dRFX target genes have not been described as being involved in ciliogenesis in any organism. *CG13415/Chibby *encodes a protein that interacts with the β-catenin protein and has been shown in *Drosophila *and in mammalian cells to antagonize the Wg/Wnt signaling pathway [63-65]. The second gene, *CG31036*, has an unknown function and no obvious ortholog in vertebrates. Protein structure prediction algorithms detect a central transmembrane domain and a signal peptide at the amino-terminus of the protein encoded by *CG31036*.

### Expression profile of three novel dRFX target genes

In order to further validate our screen, we chose three genes (*CG6129/rootletin*, *C13125/TbCMF46 *and *CG31036*) for *in vivo *study. *CG6129 *was selected to address the question of conservation in *Drosophila *of the dual role described in mammals for the rootletin protein in centrosome and ciliary biology. *CG13125 *is of particular interest to evaluate the possible involvement of a 'motility gene' in *Drosophila *sensory cilia. Last, since nothing was known about *CG31036*, we wanted to address whether this gene is involved in ciliogenesis and, thus, validate the overall X-box screening strategy.

Reporter constructs were made by cloning large promoter fragments including the conserved X-box, plus coding sequences in frame with green fluorescent protein (GFP). Transgenic flies were established and analyzed for GFP expression. Two types of ciliated cells have been described in *Drosophila*: spermatozoa and type I sensory neurons that innervate the proprioceptive chordotonal organs and external sensory organs that are mechano- or chemosensory. Remarkably, the expression of all three reporter constructs was observed only in type I sensory neurons. As a control, reporter GFP expression was compared to mRNA expression by *in situ *hybridization. CG6129/rootletin protein expression reproduces the expression of the transcript in only type I sensory neurons of the embryo (data not shown). CG31036 RNA expression is also available from the BDGP database [66]. CG31036 mRNA is restricted to type I sensory neurons of the head, thoraxes and abdomen of the embryo and reflects the protein expression of our transgene. However, we did not observe a strong protein expression in the gut as observed for the transcript. This could either reflect a non-specific hybridization signal or the presence of other transcript isoforms driven by a different promoter. We could not detect CG13125 transcripts by *in situ *hybridization, likely illustrating the faint expression of this gene in *Drosophila*.

**Figure 4:**
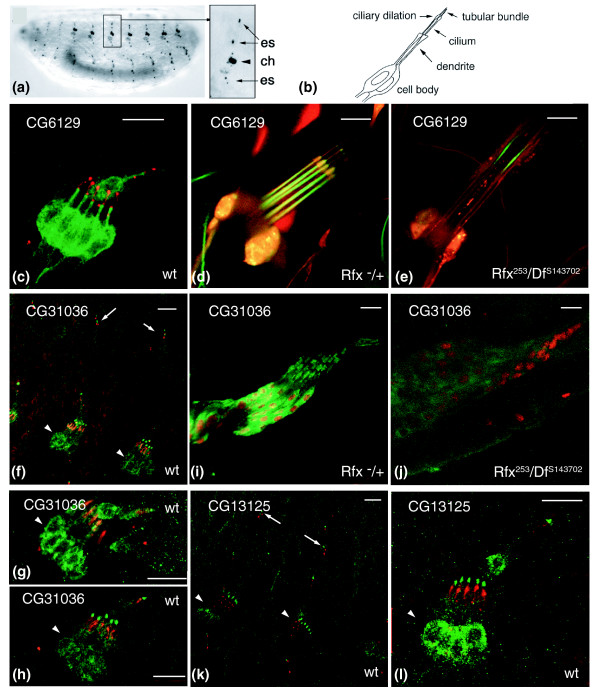
Reporter GFP expression studies for three X-box containing genes. **(a) **Stereotypical arrangement of type I sensory neurons in a *Drosophila *embryo, anterior to the left, stained with the 21A6 antibody with a magnification of the dorsal and lateral neurons of one abdominal segment as visualized in (f,k). The arrowhead indicates the five lateral chordotonal neurons (ch) and the arrows point to the neurons of the external sensory (es) organs. **(b) **Schematic of two typical chordotonal organs of *Drosophila*. **(c-l) **Confocal imaging of GFP expression of transgenic lines carrying the promoter region and coding sequences fused to the GFP for *CG6129/rootletin *(c-e), *CG31036 *(f-h) and *CG13125/TbCMF46 *(k,l). GFP expression is only observed in ciliated sensory neurons of *D. melanogaster *where the chimeric GFP proteins are localized to the ciliary apparatus. (c) *CG6129::GFP *reporter expression (green) is observed in embryonic chordotonal organs, mainly along the dendrite from the base of the cilium to the cell body. The 21A6 antibody (red, see Materials and methods) labels the ciliary dilation of the cilium. (d,e) Live GFP imaging of the lateral pentascolopidial chordotonal organs in male third instar larvae of *dRfx *deficient (e) and control (d) sibs. The *elav-RFP *expression (red) labels all neurons. *CG6129/rootletin *is regulated by *dRfx *as no GFP expression is observed in *dRfx *deficient larvae. (f-h) *CG31036::GFP *reporter expression (green) is observed both in the external sensory neurons (arrows in (f)) and the chordotonal neurons in the embryo (arrowhead in (f-h)). The 21A6 antibody (red) labels the ciliary dilation at the tip of the dendrite. CG31036::GFP protein localization appears to be slightly different depending on the fixative used (paraformaldehyde in (g), methanol in (h)). (i-j) Immunodetection of CG31036::GFP expression in leg chordotonal organs of 72-hour pupae in *dRfx *deficient (j) or control (i) sibs. The anti-ELAV antibody (red) labels all neurons. No *CG31036::GFP *expression is observed in *dRfx *deficient pupae (j). (k,l) *CG13125::GFP *expression is observed by immunodetection in the embryonic chordotonal organs (arrowheads in (k,l)) but also in the external sensory neurons (arrows in (k)). A higher magnification of the lateral chordotonal organs (l) shows that GFP is apposed to the 21A6 immunostaining (red). Scale bar = 10 μm.

Chimeric CG6129::GFP protein was present in the rootlet processes of the chordotonal dendrites, in agreement with the predicted function of rootletin in ciliary rootlet organization (Figure 4). It was also detected faintly at the cilium tip (Figure 4d) and clearly in axons (Figure 4). Since our construct does not include all the coding sequences of the rootletin protein, it is possible that the GFP expression does not reflect the exact location of the endogenous protein. Rootletin has been shown in mammalian cell culture to be localized to the ciliary rootlet and to be involved in centrosome cohesion [56]. We show that *CG6129/Rootletin *expression is restricted to ciliated chordotonal neurons in *Drosophila*, thus suggesting an involvement only in ciliogenesis. Despite strong GFP expression in the chordotonal organs, no expression was observed in the ciliated sensory neurons that innervate external sensory organs. Either the expression in those cells is too weak, or ciliary rootlets in *Drosophila*, as represented by CG6129/rootletin GFP expression, are restricted only to chordotonal organs, as observed previously by electron microscopy [67,68].

CG31036::GFP specifically marks the ciliated endings of chordotonal neurons and confirms that this novel protein is a component of ciliated endings (Figure 4). The GFP signal is apposed to the 21A6 antibody staining, directed against the eyes shut protein, which has been described to locate at the ciliary dilation around the tip of the ciliated segment [69]. This implies that CG31036::GFP most likely locates to the tip of the tubular bundle that extends after the ciliary dilation (schematic in Figure 1a). However, only ultrastructural observations of immunogold labelings will allow precise subcellular localization of both CG6129/rootletin and CG31036. Interestingly, CG31036::GFP expression is also detectable in external sensory neurons as a dot apposed to the 21A6 antibody staining (Figure 4f). Finally, we confirmed that both reporter constructs are under *dRfx *control as the GFP signal was completely shut down in a *dRfx *mutant background (compare Figure 4d and 4e or 4i and 4j).

For the third construct, CG13125::GFP localization was consistently observed in the chordotonal neurons at the base of the cilium, presumably the basal body region, and also at the tip of what is likely the cilium. GFP expression was also often observed in the external sensory neurons as a dot but without consistent reproducibility, probably illustrating a threshold level of expression for these cells and the faint level of expression of the *CG13125/TbCMF46 *transgene (Figure 4k,l).

In conclusion, the three novel dRFX target genes that we identified in our X-box motif searches are indeed under dRFX control *in vivo *and specifically expressed in ciliated sensory neurons in *Drosophila*. In addition, they encode proteins that are localized to the base or the tip of the cilium, thus suggesting a role in ciliary structure or function.

## Discussion

Ciliogenic RFX regulatory networks are conserved between *C. elegans *and *D. melanogaster*. Based on these first observations, the genomic screens we conducted combined with functional and *in vivo *gene analyses led to the identification of at least 11 novel genes that had never been described as RFX targets in any biological model. In addition, our screen allowed us to identify at least two novel genes specifically expressed in ciliated sensory neurons in *Drosophila *that are potentially involved in sensory ciliogenesis. These results validate the accuracy of our screens. Our work thus provides a new set of candidate genes for further functional studies in ciliogenesis.

### Molecular nature of RFX target gene products

Our *Drosophila *genome wide X-box screen led to the identification of 83 X-box genes among which we report 11 novel RFX targets. Combined with the genes identified by comparisons to *C. elegans *or to other genomic studies in *Drosophila *(Table 1) [13], we report 35 genes regulated by dRFX in *Drosophila*. Most of these genes can be classified based on their described function. Many of the RFX target genes are involved in IFT, which is necessary for cilium assembly and function [1]. Remarkably, a second class of genes regulated by dRFX includes all the *Drosophila *homologs of *BBS *genes. Similarly, most *C. elegans BBS *genes are regulated by DAF-19 [14,36,37]. This strong dependence of *BBS *genes on RFX control may thus be conserved in mammals. Hence, RFX proteins may be involved in BBS in humans. Interestingly, two of the three *Drosophila *genes coding for proteins with B9 domains are also controlled by dRFX (*tectonic*, *CG14870*). One human B9 domain protein, MKS1, is known to be involved in the human Meckel-Gruber syndrome [70]. The molecular function of this domain is unknown and work in *Drosophila *suggested that these two B9 domain containing proteins are likely involved in ciliogenesis [13]. Several of the novel dRFX target genes that we identified in this study encode known components of the ciliary axoneme and associated structures, such as axonemal dyneins or rootletin. Other genes encode different types of proteins likely involved in sensory transduction (*CG4536*/*osm-9*/*TRPV4 *or *MIP-T3*). A last class includes genes for which the function is either not described or poorly understood, such as *CG31036 *and *CG13125*. However, our functional studies strongly suggest that they are also probably involved in sensory ciliogenesis in *Drosophila *as well. Thus, RFX target genes play various roles in ciliary structure and function and our X-box search strategy has proven to be useful to identify novel ciliogenic genes.

### Database mining using the X-box promoter motif

This full set of dRFX target genes in *Drosophila *is of crucial importance, as we can now more precisely define X-box sequences and the promoter context required for dRFX control. This will be particularly useful for further database mining of dRFX target genes in *Drosophila*. In fact, several genes that are under dRFX control (Table 1, for example *CG4525*, *CG17599*) for which an X-box can be identified did not come out in the whole genome X-box screen. Several reasons can explain this result. First, homologs were not all annotated in CDS listings that were available at the time of the search (for example, *CG18631*, *CG9595*, *nompB *in *D. pseudoobsura*). Second, annotation of both *Drosophila *databases is incomplete, as sometimes the start codon is not properly defined for all genes. Our X-box search algorithm keeps only genes for which the X-box match is upstream of the ATG. For example, for *CG15666/GA13881*, we clearly predict that the correct ATG should be considered 75 bp downstream of the currently defined ATG, based on evolutionarily conserved sequences. This definition clearly excludes the homologous genes *CG15666 *and *GA13881 *from the dataset. However, as illustrated in Table 2, in a few cases, our X-box consensus cannot define a clearly conserved X-box match in the two *Drosophila *species for genes that appear to be down-regulated in a *dRfx *mutant, while several individual X-boxes are found separately in each organism. This could either reflect that these genes are not direct dRFX targets but are shut down by a feedback control loop that is not dependent on a X-box motif, or that the X-box is only loosely conserved in some promoter contexts. Notably, homologs of these genes in *C. elegans *are under RFX (DAF-19) control and have a well defined X-box (for example, *CG9333/che-2*, *CG13691/bbs-8*), which argues in favor of the second possibility. Interestingly, we also quantified the expression levels in control and *dRfx *deficient *Drosophila *of several genes of the DCBB dataset that did not come out of the X-box genome-wide motif search. It allowed us to identify several novel genes that are indeed down-regulated in *dRfx *mutants, but for which no conserved X-box can be recognized based on our initial consensus motif (AL, unpublished). Altogether, our observations clearly highlight the difficulties encountered in motif definition in promoters. Similar conclusions were deduced from a parallel approach performed in *C. elegans*, which has led to the identification of several novel DAF-19 target genes [38]. Interestingly, in that study the *in silico *search was associated with microarray analysis of transcripts in wild-type and *daf-19 *mutant worms. The *in silico *search allowed the identification of 93 X-box genes. Yet, among the 466 genes that were shown to be down-regulated at least two-fold in microarray hybridization experiments, only 25 were also represented in the 93 *in silico *X-box gene list. Thus, *in silico *searches on isolated motifs are likely hampered by a high level of false negatives. In order to improve the screening efficiency, the use of combinatorial motif searches would probably greatly enhance the accuracy of the screen as proposed by other studies [71,72]. Even though, since conserved X-boxes that we identified are rarely associated with highly conserved surrounding sequences (Table 2), it is reasonable to assume that other conserved nearby motifs, still to be identified, could help to discriminate between false positives and false negatives.

### Regulatory network of ciliary genes

We have identified 35 genes that are transcriptionally down-regulated in *dRfx *mutants. We show that RFX regulatory networks are conserved between *C. elegans *and *Drosophila *as most of the genes controlled by DAF-19 in *C. elegans *are also under dRFX control in *D. melanogaster*. Interestingly, our results show that only certain subsets of ciliogenic genes are regulated by RFX proteins. For example, in our assay conditions all the genes known to be involved in IFT-A complexes are not regulated by dRFX, whereas all IFT-B homologous proteins are regulated by dRFX. In addition, retrograde motors are also regulated by dRFX (*CG15148/btv *and *CG3769*), whereas anterograde motors seem not to be. Indeed, in addition to *CG10642/KIF3A*, the main described anterograde motor in several organisms, we have shown that two other kinesin subunits, *CG17461/Kif3C/osm-3 *and *CG7293/Klp68D*, are invariantly expressed in wild-type and *dRfx*-deficient *Drosophila *(AL, data not shown). It is also interesting to note that all the *BBS *gene homologs in *D. melanogaster *are under dRFX control (Table 1).

The biological significance of these observations is unclear. It could reflect the fact that IFT-B proteins, BBS proteins and the dyneins involved in IFT are dedicated to ciliogenesis and, therefore, need to be turned on concomitantly only when the cilium is formed, whereas IFT-A complexes or anterograde transport kinesin II share more complex regulatory controls as they might be necessary also for other cellular functions. This is the case for kinesin II motors [73], but does not seem to be true for IFT-A complexes as these proteins are proposed to be specific for ciliated organisms [13]. In *C. elegans*, the ciliary IFT machinery works in modular fashion [74], and it is tempting to speculate that RFX-dependent proteins could be involved in specialized ciliogenic transport modules.

Genes necessary for centriole biogenesis or replication, such as the recently described *DSas-6*, *DSas-4 *or *sak *genes [75-78] are not present in our screen and no conserved X-box can be found upstream of these genes. Thus, dRFX does not seem to regulate centriole biogenesis and appears to be restricted to cilia assembly only.

To find which transcription factors are responsible for governing other sets of ciliary proteins will certainly be one track to follow. Based on our data, it would be of particular interest to compare promoter sequences of genes, either regulated by dRFX, or not. It may allow us to discover novel regulatory motifs and protein modules that are necessary to coordinate ciliogenesis control. So far, only a few transcription factors have been shown to be involved in the control of ciliogenesis: the RFX proteins [21,23,24], Foxj1 [16], and HNF1-beta [17]. However, the last two have no obvious homologs in *Drosophila*. Thus, our work strongly suggests that novel transcription factors necessary for ciliogenesis still need to be discovered.

### Novel RFX target genes

Some of the novel RFX target genes found in *Drosophila *were unexpected. For example, we identified several proteins that are proposed to be involved in flagella or cilia motility, such as dynein heavy chains (*CG17150/Dhc93AB*). Recently, a *CG13125 *homolog has also been shown to function as a motility factor in *T. brucei *(TbCMF46) [59]. Sensory cilia are thought not to be motile in general. However, it has been shown that *Drosophila *chordotonal neurons of the antenna generate motion that depends on the integrity of proteins encoded by genes such as *CG15148*/*btv *(cytoplasmic dynein heavy chain) or *CG14620*/*tilB *(*LRRC6 *homolog), described to affect the axonemal structure [52,79] (D Eberl, personal communication). In addition, cilia of the chordotonal neurons of the grasshopper bend upon vibration stimulation [80]. Thus, proteins involved in axonemal motility might be important for motion generation of the cilium in response to mechanical stimulation. It will be of high interest to determine whether flies defective in these 'motility' genes are affected in hearing and, more specifically, in the motility of the mechanosensory cilium that amplifies hearing vibrations. Interestingly, *CG13125/TbCMF46 *does not seem to be expressed in fly testis (AL, unpublished), where the spermatozoa are the only cell type with a motile flagellum in flies. This suggests that like *CG15148/btv*, *CG13125/TbCMF46 *function could be restricted to the sensory cilium and, more specifically, in allowing these cilia to mechanically respond to auditory vibrations [52]. Thus, our data suggest that in the fly, possible axonemal motility could be regulated by different subsets of proteins in sperm flagella and in mechanosensory cilia. This is of particular interest with regard to hearing in mammals, which is dependent on hair cell motility. It will be very interesting to determine whether the *CG13125/TbCMF46 *homolog in mammals does have a specific function in those cell types.

We also identified in our screen three genes (*CG6054/Su*(*fu*), *CG13415/Cby*, *CG33038/Ext*(*2*)) known to be involved in the *hedgehog *or *wingless *signaling pathways in *Drosophila*. *Su*(*fu*) and *Ext*(*2*) are involved in the Hedgehog pathway and Su(fu) is localized to cilia in mammalian cells [81]. However, *Su*(*fu*) and *Ext*(*2*) do not appear to be under *dRfx *control according to real-time PCR quantification results (Table 3) and may be false positives in our screen. This result argues in favor of the generally accepted observation that the Hedgehog signaling pathway does not seem to depend on ciliogenic proteins in *Drosophila *[82]. Only *Chibby *(*Cby*) is statistically down-regulated two-fold in a *dRfx *deficient background. *Cby *was isolated in a two-hybrid screen for armadillo/beta-catenin interactors. RNAi knock-down of *Cby *in *Drosophila *embryos leads to ectopic activation of the wingless pathway [63]. *Cby *is also described to antagonize the Wnt/beta-catenin pathway in mammalian cells [64,65]. However, the expression pattern of *Cby *in *Drosophila *is not documented, so we do not know if the variations of expression observed in the *dRfx *deficient background are connected to *dRfx *expression and, thus, if it is biologically significant.

Among the 83 genes with conserved X-boxes between *D. melanogaster *and *D. pseudoobscura *(Table 3), several genes were hardly detectable by quantitative RT-PCR. Hence we were unable to determine by this approach if they are under dRFX control. This could reflect that these genes are expressed only in a subset of sensory neurons and, thus, difficult to detect by quantitative RT-PCR. Nevertheless, several genes are interesting as potential ciliogenic or RFX target genes. For example, *CG14079 *is homologous to a mouse protein that appears to be specific to testis. *CG11356 *is homologous to mammalian *arl13*, which has just been isolated in an ethyl-nitroso-urea screen for neural tube defects in mouse. Indeed, mutation of *arl13 *affects ciliary architecture and Sonic-Hedgehog signaling in mouse [83]. This gene, *CG11356*, was not found in any previous ciliogenesis study, again illustrating the accuracy of our screen. Functional studies in *Drosophila *will be of particular importance to demonstrate the role of this gene in sensory ciliogenesis.

## Conclusion

We have identified more than 30 dRFX target genes in *Drosophila *by exploiting the efficiency of the X-box promoter motif search by using two divergent *Drosophila *species in a comparative approach. These full sets of RFX dependent or independent ciliary genes are of particular importance for studies of X-box promoter motifs and associated promoter contexts in *Drosophila*. More remarkably, our screen allowed the identification of at least two novel genes specific to sensory ciliary architecture in *D. melanogaster *and provides several new RFX target gene candidates potentially involved in ciliogenesis. This is of particular importance with regard to the growing number of human diseases that are being associated with ciliary defects (for reviews, see [4,5,7]).

## Materials and methods

### Quantitative RT-PCR

Total RNA was extracted from 40-hour old puparium using TRIzol reagent (Invitrogen, Carlsbad, CA, USA) or RNeazy (Qiagen, Venlo, The Netherlands). Pupae head and abdomen were removed as well as internal organs and muscles in order to enrich as much as possible the extract for sensory organs from thoraxes, legs and wings. DNA was digested with DNA-free reagent (Ambion, Austin, TX, USA). Reverse transcription (RT) was performed on 2 μg of RNA derived from pools of 5 thoraxes with random hexamers (Promega, Madison, WI, USA) with RevertAid™ H Minus M-MuLV reverse transcriptase (Fermentas, Burlington, Canada). Real-time PCR analysis was performed with SYBR Green fluorescent PCR (Qiagen) in a LightCycler (Roche, Basel, Switzerland) or a MX3000 (Stratagene, Cedar Creek, TX, USA) fluorescent temperature cycler. Primer sequences specific for each gene are available upon request. Primers were used at 0.5 μM. PCR conditions were as follows: 95°C, 15 minutes; 35 × (95°C, 15 s; 60°C, 20 s; 72°C, 20 s). According to melting point analysis, only one PCR product was amplified under these conditions. RNA extracted from wild-type samples was used to generate a standard quantification curve for each gene, allowing the calculation of relative amounts of transcripts in mutant samples compared to wild type. All reactions were performed with four biological replicates and two technical replicates. Results were normalized with respect to *CG9874/TBP *expression and standard errors of the mean were calculated. Results are expressed as relative mutant to wild-type expression ratios. Significance levels were tested with unpaired *t*-test.

### Bioinformatics

Individual X-boxes (consensus RYYNYYN{1-3}RRNRAC) were searched for in the 5' upstream regions of ATGs on the same strand (+) and the antiparallel strand (-) in both *D. melanogaster *and *D. pseudoobscura *homologs [84]. Genome wide searches for X-box promoter motifs were primarily performed using a Perl-based algorithm that identifies all possible matches in a given DNA sequence. First, the algorithm finds all sequences that match a defined consensus, then the main module implements a cross-match file that compares a 3 kb window downstream of each match to a file containing the DNA sequences for all predicted genes [36]. Genome sequence information, gene prediction and CDS files for X-box searches were obtained from the following sources: the *D. melanogaster *complete genome sequence used was BDGP release 4; the complete CDS list was built from release 3.2.1 [85]. For *D. pseudoobscura *the 28 August 2003 genome assembly was used and release 2.1 of CDS sequences from BCM-HGSC were used [40]. Reverse BLASTP analysis was performed between the two CDS files in order to establish a list of orthologous genes between the two fly species with a cut-off value of BLAST e-score <1 e^-10^. Comparisons of all listed gene information were performed on a Unix platform. BDGP and Flybase databases were mined for expression patterns and gene information. Genome conservation between the two fly species was evaluated using the VISTA interface [86].

### DCBB dataset

The ciliary and basal body genes in Additional data file 2 were identified using a reverse BLASTP strategy to define the best homologous proteins or genes described in the following studies: 210 proteins published in Table 2 from the human ciliary proteome [10] as modified by Marshall [87], 159 putative target genes of DAF-19 [36], 219 over expressed genes after deflagellation in *C. reinhardtii *described in Table 9 of Stolc *et al*. [15], 54 genes (Table 1) expressed in ciliated sensory neurons in *C. elegans *[37], 654 proteins identified in *C. reinhardtii *flagella [11], 380 proteins identified in the *T. brucei *flagella proteome [12] and 114 proteins listed in Table S1 for the human cell centrosome [44]. The following *Drosophila *homologs were extracted from published work: 260 genes described as homologous to the FABB proteins from *C. reinhardtii *in Table S1 of Li *et al*. [14], 51 genes described as homologous to 195 proteins described in Table S2 for the basal body proteome of *C. reinhardtii *[45] and 187 genes from Table S1 of compartmentalized cilia predicted genes, which has been modified to 188 genes according to Flybase annotation [13].

### Reporter constructs

DNA fragments were amplified from wild-type fly genomic DNA using the Expand Long Template PCR system (Roche). Cloning strategies used primers to clone in frame the gene of interest to the GFP sequence of the PW8-GFP vector [88]. CG13125::GFP plasmid, a 3,547 bp genomic DNA fragment containing the complete coding sequence of CG13125-RA and RB, was amplified from Canton-S using primers starting 1,484 bp upstream of the RB ATG until the penultimate codon of the gene. CG6129::GFP plasmid, a 4,129 bp genomic DNA fragment containing part of the CG6129-RB gene, was amplified by PCR from Charolles genomic DNA using primers starting 2,619 bp upstream of the RB ATG. CG31036::GFP plasmid, a 3,780 bp genomic DNA fragment containing part of the CG31036-RA gene, was amplified by PCR from Canton-S using primers starting 1,800 bp upstream of the ATG. All coding regions cloned were entirely sequenced prior to transgenesis. Transgenic lines were established by P-element mediated germline transformation as described [89].

The following fly stocks were used for experiments: P{mecCP:Gal4^37*a*1^, P{Osm-1:Gal4}^T17#7a1^, P{CG9227:Gal4}^T32#10a2 ^and P{CG3259:Gal4}^T39#13a1 ^were gifts from Tomer Avidor-Reiss (Harvard Medical School, Boston, MA, USA). P{UAS-RFP}^31 ^was a gift from Maurice Kernan (Stony Brook University, New-York, NY, USA) and P{GawB:elav}^C155 ^and P{UAS-mCD8::GFP.L}^LL6 ^strains were provided by the *Drosophila *Bloomington Stock Center, IN, USA.

### Fly genetics and observations

Fly genotypes used to extract RNA were *st dRfx*^253^*e ca/Df*^*S*143702 ^for mutants and *st e ca/Df*^*S*143702 ^for control flies [23]. Control and mutant flies presented in Figure 1 share the same genotype with the exception of the third chromosome, which is heterozygous for the *dRfx*^253 ^carrying chromosome in the controls. Genotypes for flies were: oseg1 flies, *y w P{UAS:CD8:GFP}/Y; P{mecCP:Gal4*^37*a*1^*}/+; P{UAS:CD8:GFP} Rfx*^253^*/Rfx*^49^; osm-1 flies, *w; P{Osm-1:Gal4*^*T*17#7*a*1^*}/+; P{UAS:CD8:GFP} Rfx*^253^*/Rfx*^49^; CG3259 flies, *y w P{UAS:CD8:GFP}; st Rfx*^253^*e P{CG3259:Gal4*^*T*39#13*a*1^*}/Rfx*^49^; and CG9227 flies, *y w P{UAS:CD8:GFP}/Y; st Rfx*^253^*P{CG9227:Gal4*^*T*32#10*a*2^*}/Rfx*^49^. Control and mutant flies presented in Figure 4 were sibs sorted from the same crosses. For *CG6129 *expression in a *dRfx *deficient background, females (*w elav*^*c*155^*P{UAS-RFP*^31^*}; Rfx*^49^*/TM6B*, *Tb*) were crossed with males (*w;P{CG6129:EGFP}*^*M*33^*; Rfx*^253^*/TM6B*, *Tb*). For CG31036, the crosses were: females (*w; P{CG31036:EGFP}*^*F*27^*; Df*^*S*143702^*/TM6B*, *Tb*) with males (*w; P{CG31036:EGFP}*^*F*27^*; Rfx*^253^*/TM6B*, *Tb*).

The preparation of embryos for staining assays was carried out according to general methods described previously [90]. Live observations of dechorionated embryos and larvae were performed on mounted material under coverslips in DakoCytomation media. For pupae immunostaining, 72- to 96-hour old animals were fixed for 20 minutes in 4% paraformaldehyde, 3% triton X-100 in phosphate-buffered saline. Primary antibodies were rabbit anti-GFP (1:250) from Torres Pines Biolabs (Houston, TX, USA), or (1:500) from Molecular Probes (Invitrogen, Carlsbad, CA, USA), mouse anti-eys 21A6 and mouse anti-Futch 22C10 (kindly provided by S Benzer), mouse anti-elav 9F8A9 (1:500) obtained from the Developmental Studies Hybridoma Bank, Iowa City, IA, USA. Secondary conjugated antibodies were A488 and A546-anti-mouse and anti-rabbit (Molecular Probes, Invitrogen, Carlsbad, CA, USA). Images were obtained on a Zeiss Imager Z1 and LSM510 confocal microscope.

## Abbreviations

BBS, Bardet Biedl syndrome; bp, base pair; CDS, coding sequence; DCBB, *Drosophila *cilia and basal body; IFT, intraflagellar transport; RFX, regulatory factor X; TRP, transient receptor potential.

## Authors' contributions

A.L. and B.D. designed the experiments, wrote and revised completed this manuscript; A.L., E.E. and P.S. performed genome wide X boxes search. A.L., R.D. and G.G. realized the identification of the first set of target genes. A.L., R.B., V.R. and E.C. carried out fly genetic experiments. A.L., V.R. and R.B. performed cytology.

## Additional data files

The following additional data are available with the online version of this paper. Additional data file 1 is a table listing the full set of 412 X-box genes conserved between *D. melanogaster *and *D. pseudoobscura*. Our X-box search across *D. melanogaster *and *D. pseudoobscura *species identified 412 genes with a conserved X-box both in sequence and distance upstream of the ATG of homologous genes between the two fly genomes. Additional data file 2 is a table of the DCBB genes list established for *D. melanogaster*. Genes presented in this table are homologous to proteins identified as putative or confirmed ciliary or basal body components. They are sorted as follows: a first group of genes with annotated molecular functions, a second group of genes for which homologs in vertebrates have been reported, a third group of genes with no vertebrate homolog. Each category is sorted by the number of studies reporting each gene or its homolog. Additional data file 1 is a table listing the number of *Drosophila *genes homologous to ciliary genes identified in previously published studies.

## Supplementary Material

Additional data file 1 Our X-box search across *D. melanogaster *and *D. pseudoobscura *species identified 412 genes with a conserved X-box both in sequence and distance upstream of the ATG of homologous genes between the two fly genomes. Click here for file

Additional data file 2 Genes presented in this table are homologous to proteins identified as putative or confirmed ciliary or basal body components. They are sorted as follows: a first group of genes with annotated molecular functions, a second group of genes for which homologs in vertebrates have been reported, a third group of genes with no vertebrate homolog. Each category is sorted by the number of studies reporting each gene or its homolog. Click here for file

Additional data file 3 Number of *Drosophila *genes homologous to ciliary genes identified in previously published studies. Click here for file
