# Supplementary material for: Identification of novel regulatory factor X (RFX) target genes by comparative genomics in Drosophila species
Source: Genome Biol. 2007 Sep 17;8(9):R195. doi: 10.1186/gb-2007-8-9-r195 (PMC2375033; doi:10.1186/gb-2007-8-9-r195)
Supplement: Additional data file 3 — Number of Drosophila genes homologous to ciliary genes identified in previously published studies. [file gb-2007-8-9-r195-S3.doc]

Table S3. Number of Drosophila genes homologous to ciliary genes identified in previously published studies.

*: Table S2

|  | *N° of homologous genes in* Drosophila melanogaster | | | |
| --- | --- | --- | --- | --- |
| Study | DCBB  Drosophila Cilia & Basal Body* | Ciliome  database [49] | Ciliary proteome database [48] | |
|  |  |  | R | Nr e-^10^ |
| Ostrowski *et al.* [10] | 126 | 72 | 74 | 91 |
| Avidor-Reiss *et al.* [13] | 188 | 187 | 161 | 172 |
| Li *et al.* [14] | 260 | 186 | 276 | 392 |
| Blacque *et al.* [37] Xbox (+ SAGE) | ­50 | 727 (+656) | 187 | 298 |
| Efimenko *et al.* [36] | 117 | 168 | 188 | 327 |
| Pazour *et al.* [11] | 192 | 253 | 202 | 280 |
| Stolc *et al.* [15] | 88 | 62 | 86 | 108 |
| Broadhead *et al.* [12] | 69 | - | 83 | 127 |
| Andersen *et al.* [44] | 56 | - | 98 | 99 |
| Keller *et al.* [45] | 51 | - | 40 | 52 |
| *D. m.* Total | 815 | 1747 | 759 | 1075 |
| ***D. m.* non redundant** | **815** | **1736** | **692** | **907** |
